# High miR-205 expression in normal epithelium is associated with biochemical failure - an argument for epithelial crosstalk in prostate cancer?

**DOI:** 10.1038/s41598-017-16556-2

**Published:** 2017-11-24

**Authors:** Yngve Nordby, Elin Richardsen, Nora Ness, Tom Donnem, Hiten R. H. Patel, Lill-Tove Busund, Roy M. Bremnes, Sigve Andersen

**Affiliations:** 1 Department of Clinical Medicine, The Arctic University of Norway, Tromso, Norway; 2 Department of Urology, University Hospital of North Norway, Tromso, Norway; 3 Department of Oncology, University Hospital of North Norway, Tromso, Norway; 4 Department of Clinical Pathology, University Hospital of North Norway, Tromso, Norway; 5 Department of Medical Biology, The Arctic University of Norway, Tromso, Norway

## Abstract

Due to insufficient prognostic tools, failure to predict aggressive prostate cancer (PC) has left patient selection for radical treatment an unsolved challenge. This has resulted in overtreatment with radical therapy. Better prognostic tools are urgently warranted. MicroRNAs (miRs) have emerged as important regulators of cellular pathways, resulting in altered gene expressions. miR-205 has previously been observed downregulated in PC, acting as tumor suppressor. Herein, the expression of miR-205 in prostate tissue was examined in a large, well-described cohort of 535 Norwegian prostatectomy patients. Using *in situ* hybridization, miR-205 expression was semiquantatively measured in normal and tumor tissues from radical prostatectomy specimens. Associations with clinicopathological data and PC relapse were calculated. Expression of miR-205 was lower in tumor epithelium compared to normal epithelium. No association was observed between miR-205 expression in primary tumor epithelium and cancer relapse. In contrast, high expression of miR-205 in normal epithelium was independently associated with biochemical relapse (HR = 1.64, p = 0.003). A prognostic importance of miR-205 expression was only found in the normal epithelium, raising the hypothesis of epithelial crosstalk between normal and tumor epithelium in PC. This finding supports the proposed novel hypothesis of an anti-cancerogenous function of normal epithelium in tumor tissue.

## Introduction

Prostate cancer (PC) is the most common malignancy in men^1^. The majority of prostate tumors is detected at early stages with uncertain prognosis. Prognostic factors like prostate specific antigen (PSA) and histologic scores are well established. These are, however, imprecise and fail to accurately predict PC outcome. This has led to a significant overtreatment with radical therapy (prostatectomy or radiation), while most patients probably would have managed better without treatment^2–5^. Side effects and lack of benefit for costly treatment is discrediting aggressive treatment. However, high incidence and uncertain prognostication makes PC the second most common cause of cancer death in men^6^. Thus, there is an urgent need for better prognostic tools to aid treatment selection, in the interest of both patients and the public.

The micro-RNAs (miRs) are small noncoding RNAs regulating protein expression and numerous cellular processes^7^. These are involved in the normal functioning of cells, while dysregulations of miRs are associated with disease. Various dysregulations of certain miRs (oncomirs) associated with specific cancers have been identified, and they may have either tumor suppressor or oncogenic functions able to modulate nearly all stages of cancer progression including proliferation, apoptosis, cell migration, angiogenesis and stem cell maintenance^8–10^. miR-205 acts either as an oncogene or as a tumor suppressor by facilitating or repressing tumor initiation and proliferation depending on type of cancer and stage^11^. Recently, there has been a major effort to target these noncoding RNAs therapeutically, and a few miRs have entered preclinical and clinical trials^12^.

While studies have demonstrated that miR-205 in general is involved in both normal development and cancer, the prognostic role of miR-205 in PC is not unambiguously clarified in PC^13,14^. miR-205 is found to be downregulated in PC tissue compared to benign tissues, and loss of miR-205 seems to be associated with an invasive phenotype and poor clinical outcome^15^. miR-205 has a tumor suppressive function by inhibiting the transition from epithelial to mesenchymal tissue, cell migration and invasion in the prostate^16^. In a recent study carried out in PC clinical samples, miR-205 was demonstrated to act against tumor initiation and progression by basement membrane maintenance or repressing the mitogen-activated protein kinase and androgen receptor-signaling pathway^17^. However, high miR-205 expression has also been associated with adverse outcome in PC patients^14^.

Since miR-205 was consistently downregulated for a selected group of 14 PC patients with rapid biochemical failure in our previous screening array of 1435 miRs in tumor tissue^18^, we set out to investigate the prognostic role of miR-205 in our large and well-described cohort of 535 Norwegian prostatectomy patients with extensive follow-up.

## Materials and Methods

### Patients

671 patients who underwent radical prostatectomy with curative intent for prostatic adenocarcinoma from 1995 to 2005, were retrospectively identified from the respective Departments of Pathology associated with the University Hospital of Northern Norway (n = 267), Nordland Hospital (n = 63) and St. Olavs Hospital (n = 330) and Levanger Hospital (n = 11). Of these, 136 patients were excluded due to (i) previous non-superficial cancer within five years of PC diagnosis (n = 4), (ii) radiotherapy to the pelvis prior to surgery (n = 1), (iii) inadequate paraffin-embedded tissue blocks (n = 130), and (iv) lack of follow-up data (n = 1), leaving a total of 535 eligible patients in the cohort. None of the patients had received pre-operative hormonal therapy. The cohort is thoroughly described in a previous paper^19^.

We collected relevant data from medical journals involving: Demographical data, age at surgery, previous medical history, retropubic or perineal surgery, and preoperative serum PSA level measured immediately before surgery. Further, we collected outcome data until the last follow-up date (December, 2015) or until patients’ death. The surviving patients’ disease-specific outcomes were recorded for a median follow-up of 12.4 years (range 1.5–20 years). These data included postoperative PSA values and postoperative therapy (radio-, hormonal- and/or chemotherapy). The following endpoints were used: Biochemical failure (BF) defined as postoperative PSA ≥ 0.4 or intervention with salvage therapy; Clinical failure (CF) defined as clinically palpable tumor recurrence in the prostate bed or metastasis verified by radiology; and Prostate cancer specific death (PCD), defined as death caused by PC stated in the patients’ journal.

### Tissues and tissue microarray construction

Tumor tissues, consisting of formalin-fixed paraffin-embedded (FFPE) blocks of prostate tissue from the patients’ prostatectomies, were collected from the archives of the pathological departments. An experienced pathologist (E.R.) re-evaluated the prostate samples and classified them according to the updated WHO guidelines^20,21^. Two pathologists (E.R. and L.T.B.) identified the most representative areas of cancer epithelium cells and adjacent stroma. Each area was sampled with at least two 0.6 mm cores. The cores were arranged in tissue microarray (TMA) blocks for large-scale analysis. Multiple 4 µm TMA sections were cut with a Micron microtone (HM355S). The detailed methodology has been reported previously^22^.

### *In situ* hybridization (ISH)

Chromogen *in situ* hybridization (cISH) was performed on Ventana Discovery Ultra instrument. Buffers and detection reagents were purchased from Roche and Labeled locked nucleic acid (LNA) modified probes from Exiqon, (hsa-miR-205-5p, No. 18099-15), positive control (U6 hsa/mmu/rno, No.99002-15) and negative control (scrambled-miRNA, No. 99004-15) were used. Positive and negative tissue controls for miR-205 comprised of a stained TMA multi-organ block. The controls comprised 12 different organs with both normal and tumor tissues. Hybridization, stringent wash temperatures and concentrations were optimized for each probe. Elix RNAse-free water was used during the process to minimize the risk of RNA degradation.

4 µm FFPE TMA slides were dried overnight at 59 °C to attach cores to Super Frost Plus slides. To ensure good distribution of reagents and to protect sections from drying, LCS (Liquid Coverslip oil, Roche 650–010) was added to all incubations in Discovery. Sections were deparaffinized in EZ Prep (Roche 950–100) at 68 °C (3 × 12 min). Heat mediated pretreatment was done at 95 °C with CC1 (Roche 950–500), 40 min for hsa-miR-205 and 24 min for scrambled miRNA. A combination of heat mediated and enzymatic pretreatment was done for U6, CC1 for 8 min at 95 °C and Protease III (Roche 760–2020) for 16 min at 37 °C. Probe concentrations were 25 nM for miR-205, 10 nM for scrambled miRNA, and 0.5 nM for U6. Denaturation was set to 8 min at 90 °C for all sections. Hybridization was performed for 60 min at 50 °C for miR-205, 57 °C for scramble miRNA and 55 °C for U6. Stringent washes were done 2 × 8 min with 2.0X RiboWash. Sections were blocked with alkaline phosphatase (AP) anti-DIG (Roche 760–4825) and were incubated for 20 min at 37 °C for immunologic detection. The enzymatic reactions was carried out with NBT/BCIP (CromoMap Blue kit, Roche (760–161) for 20 min at 37 °C. Finally, sections were counterstained and mounted.

### Scoring of *in situ* hybridization and cutoff

An experienced pathologist (E.R) and one Ph.D.-student/surgeon-in-training (Y.N) independently and semiquantatively scored viable parts of each anonymized core by light microscopy. The scorers were blinded for each other’s score. Intraclass correlation was calculated to assess agreement between the two observers. miR-205 expression was assessed and scored in tumor epithelium, normal epithelium and stroma. Each core was scored by the dominant intensity of staining: 0 = no staining; 1 = weak staining; 2 = moderate staining; 3 = strong staining, and classified as either normal or tumor epithelium. The core was scored as “missing” if the core was missing or the tissue was considered of insufficient quality to score. A final score from tumor epithelium and normal epithelium for each patient was calculated using the mean values of the observers’ scoring of the patients cores. Scoring of IHC cores were dichotomized into low and high expressions. Cut-off values were set at median to secure reproducibility and statistically sufficient numbers in each group. There was no significant difference in outcome regarding the choice of mean or median as cut-off value, hence median was preferred to avoid the influence of extreme values.

### Statistical methods

SPSS 23.0.0.0 (Chicago, IL) was used for all statistical analyses. Correlations were analyzed using Spearman’s rank correlation coefficient. Mean ranks of expressions between different tissues were compared by using the non-parametric Wilcoxon signed rank test. Univariate survival curves were drawn by the Kaplan-Meier method, and the statistical significance between survival curves was assessed by the log-rank test. Presentations of the survival curves were terminated at 194 months due to less than 10% of patients at risk after this point. For multivariate analyses, the backward conditional Cox-regression analysis was used with a probability for stepwise entry at 0.05 and stepwise removal of 0.10. A p < 0.05 was considered statistically significant for all analyses.

### Ethics

The reporting of clinicopathological variables, survival data and biomarker expressions was conducted in accordance with the REMARK guidelines. This study has been approved by The Regional Committee for Medical and Health Research Ethics, REK Nord, project application 2009/1393, including a mandatory reapprovement January 22, 2016. REK Nord waived the need for patient consent for this retrospective study. The Data Protection Official for Research (NSD) approved the establishment of the database.

## Results

### Clinicopathological variables and patient characteristics

The patients’ clinicopathological data are presented in the first part of Table 1. Gleason score was converted to the standards of the new International Society of Urological Pathology 2014 Grades (ISUP Grade) terminology^23^. The validated score for prediction of outcomes after radical prostatectomy, Cancer of the Prostate Risk Assessment Postsurgical Score (CAPRA-S Score), was calculated based on PSA, Gleason, surgical margin, extracapsular extension, seminal vesicle invasion and lymph node invasion^24,25^. Median age at surgery was 62 (47–75) years. At the last follow-up, 37% of the patients had BF, 11% had CF and 3.4% were dead of PC. Median preoperative serum PSA was 8.8 (range 0.7–104) and the median tumor size was 20 mm (2.0–50). Mean follow-up time was 12.4 years.

**Table 1:** Patient characteristics, clinicopathological variables and expressions of miR-205 and their associations with biochemical failure in 535 prostate cancer patients (univariate analyses; log-rank test, unadjusted Cox proportional hazard ratios).

Characteristics	Patients		Biochemical failure		
	(n)	(%)	5 year EFS (%)	HR (95% CI)	p
**Age**					**0.237**
≤65 years	357	67	77	1	
>65 years	178	33	70	1.19 (0.89–1.59)	
**pT-stage**					<**0.001**
pT2	374	70	83	1	
pT3a	114	21	61	2.30 (1.67–3.15)	
pT3b	47	9	43	4.41 (3.01–6.47)	
**Preop PSA**					<**0.001**
PSA < 10	308	57	81	1	
PSA > 10	221	42	68	1.65 (1.24–2.18)	
Missing	6	1	—		
**ISUP Grade**					<**0.001**
1 (Gleason 3 + 3)	183	34	83	1	
2 (Gleason 3 + 4)	219	41	77	1.35 (0.95–1.92)	
3 (Gleason 4 + 3)	81	15	70	2.14 (1.41–3.26)	
4 (Gleason 4 + 4)	17	4	58	3.14 (1.59–6.19)	
5 (Gleason ≥9)	35	6	37	4.30 (2.63–7.03)	
**Positive surgical margin**					**0.049**
No	249	47	81	1	
Yes	286	53	69	1.33 (1.00–1.76)	
**Apical positive surgical margin**					**0.063**
No	325	61	74	1	
Yes	210	39	77	0.76 (0.56–1.02)	
**Non-apical positive surgical margin**					<**0.001**
No	381	71	82	1	
Yes	154	29	57	2.25 (1.69–2.97)	
**CAPRA-S Score**					<**0.001**
0–2	169	32	88	1	
3–5	258	48	78	1.85 (1.25–2.73)	
6–12	102	19	46	5.28 (3.51–7.93)	
NC due to missing PSA	6	1	—		
**Tumor size**					<**0.001**
0–20 mm	250	47	83	1	
>20 mm	285	53	68	1.79 (1.34–2.39)	
**Perineural infiltration**					**<0.001**
No	401	75	80	1	
Yes	134	25	60	2.16 (1.63–2.88)	
**Lymphovascular infiltration**					<**0.001**
No	492	92	77	1	
Yes	43	8	47	2.26 (1.29–3.41)	
**Surgical procedure**					**0.466**
Retropubic	435	81	77	1	
Perineal	100	19	68	1.14 (0.81–1.60)	
**miR-205 in epithelium**					**0.003**
Low expression	220	41	81	1	
High expression	245	46	78	1.61 (1.18–2.21)	
Missing	70	13	—		

Abbreviations: EFS = event free survival in months; HR = hazard ratio; NC = not computable.

### Expressions

**Figure 1:**
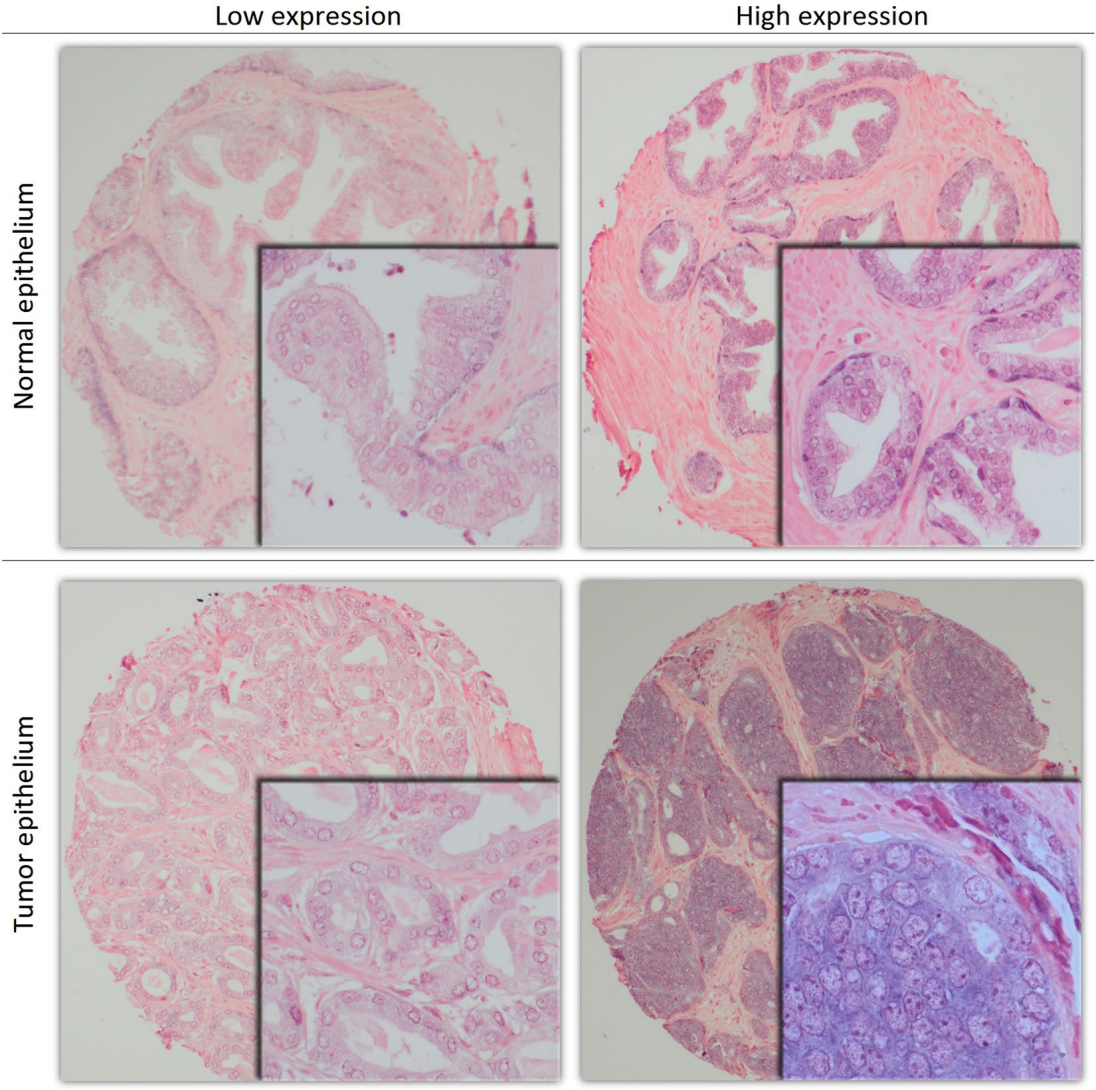
Examples of high and low expression of miR-205 in tissue microarray cores of prostate cancer tissue. 100x (main) and 400x (embedded) magnification.

Figure 1 shows examples of high and low expression of miR-205. miR-205 was expressed in both normal and tumor epithelium, where expression in tumor epithelium (mean score = 1.79) was lower when compared to normal epithelium (mean score = 1.85, p = 0.008). There was no expression of miR-205 in stroma. There was a significantly higher expression of miR-205 in normal epithelium for patients that suffered BF (mean score = 1.99) compared to patients without BF (mean score = 1.77, p = 0.001). No difference in miR-205 expression in tumor epithelium was observed comparing patients with or without BF. For validation, miR-205 staining of the multi control TMA block was compared to previous known expression profiles of different tissues^26–29^. miR-205 expression in the TMA multi control tissues was expressed negative or positive according to previous known miR-205 expression profiles.

### Correlations

The intraclass correlation coefficient between the two scorers was 0.86 (CI = 0.82–0.89, p < 0.001). None of the clinicopathological variables correlated to (r < 0.2) expression of miR-205 in tumor or normal epithelium. miR-205 expression in tumor epithelium correlated significantly to expression in normal epithelium (r = 0.27, p < 0.001).

Correlations between miR-205 expression and expressions of previously analyzed angiogenic markers [(VEGF-A, VEGF-C, VEGFR-2 and VEGFR-3) and (PDGF-B, PDGF-D and PDGFR-β)] were calculated^30,31^. miR-205 in tumor epithelium correlated to PDGF-D in tumor epithelium (r = 0.41, p < 0.001), PDGF-B in tumor epithelium (r = 0.22, p < 0.001), PDGFR-β in stroma (r = 0.21, p < 0.001), VEGF-A in epithelium (r = 0.18, p < 0.001), VEGF-C in epithelium (r = 0.25, p < 0.001) and VEGFR-2 in epithelium (r = 0.23, p < 0.001). miR-205 in normal epithelium correlated to PDGF-D in normal epithelium (r = 0.29, p < 0.001) and PDGF-B in normal epithelium (r = 0.24, p < 0.001).

### Univariate analyses

**Figure 2:**
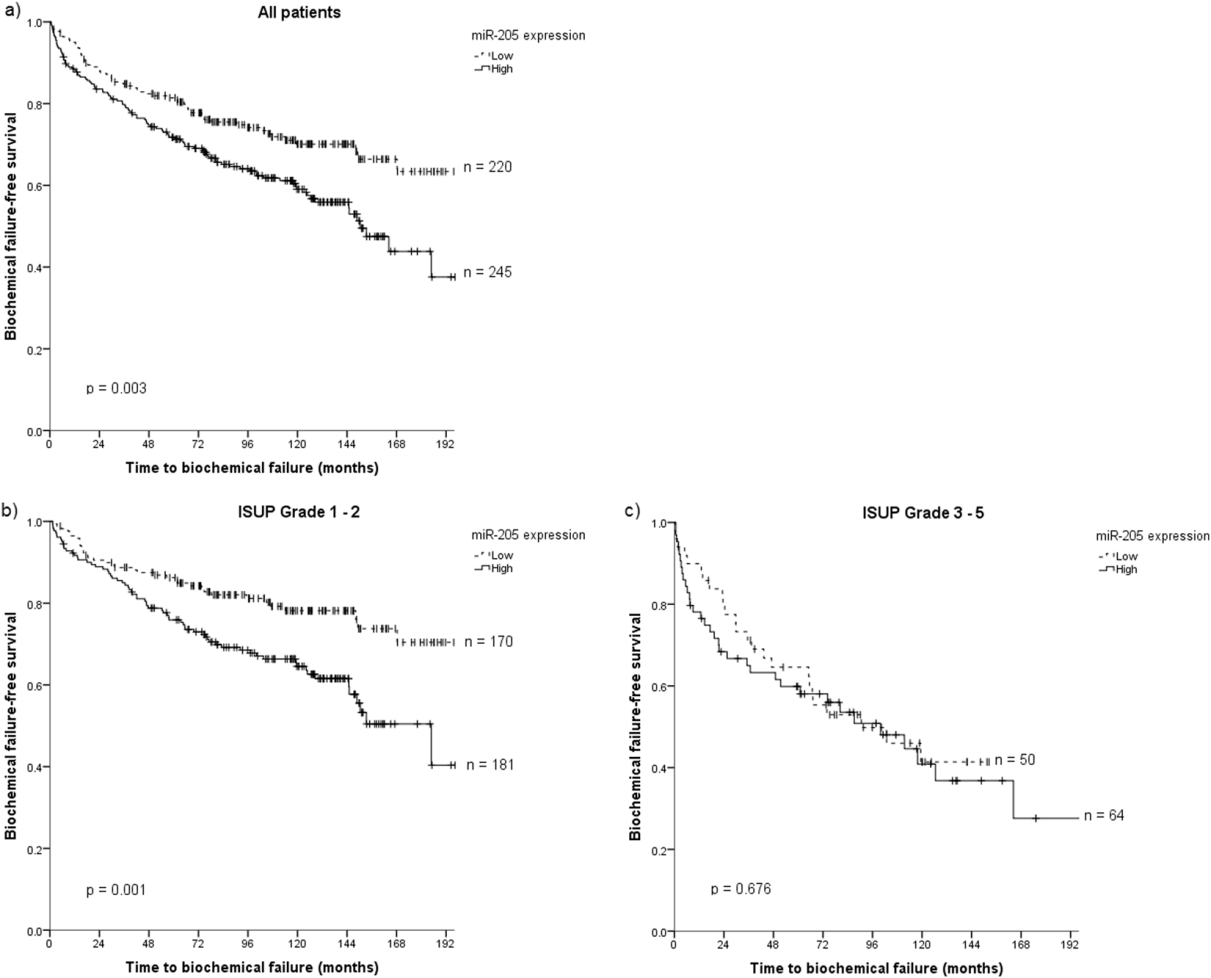
Kaplan-Meier curves of high and low miR-205 expression in normal epithelium in tissue microarray cores of prostate cancer tissue for (**a**) all patients, (**b**) patients with ISUP Grade 1 or 2 and (**c**) patients with ISUP Grade 3–5.

Results from the univariate analyses of the clinicopathological variables are presented in Table 1. The significant prognostic clinicopathological factors for BF were pT-stage (p < 0.001), preoperative PSA (p < 0.001), ISUP Grade (p < 0.001), positive surgical margin (p = 0.049) with its subclass non-apical margin (p < 0.001), CAPRA-S Score (p < 0.001), tumor size (p < 0.001), perineural infiltration (p < 0.001) and lymphovascular infiltration (p < 0.001). Significant prognostic factors for CF and PCD were previously reported^30,31^. Regarding the miR-205 biomarker, we found no association between expression in tumor epithelium and endpoints for any cut-offs (for mean cut-off and BF: p = 0.864). In contrast, high expression of miR-205 in normal epithelium was associated with BF (p = 0.003). There was a trend towards association between high miR-205 and CF, but the association was not significant (p > 0.100). For PCD, no significant outcome difference was observed regarding high or low miR-205 expression subgroups for any cut-off. A Kaplan-Meier survival curve of miR-205 expression versus BF for all patients is presented in Fig. 2.

Survival analyses for BF stratified according to clinicopathological factors were calculated to explore if there were possible subgroups where expression of miR-205 had a particular significant impact on prognosis. For patients with ISUP Grade 1 or 2 (Gleason 3 + 3 or 3 + 4), there was a significant association between BF and high miR-205 expression [n = 351, HR 1.94 (95% CI = 1.30–2.91), p = 0.001]. But no significant association was observed between the biomarker and BF in patients with ISUP Grade 3 (Gleason 4 + 3) or higher [n = 114, HR = 1.12 (95% CI = 0.66–1.88), p = 0.676]. A Kaplan-Meier survival plot of miR-205 and BF stratified on ISUP Grade is shown in Fig. 2. Regarding the post-prostatectomy outcome predictor CAPRA-S Score, there was a significant association between high miR-205 expression and BF for patients with CAPRA-S Score 0–5 [n = 374, HR = 1.75 (95% CI = 1.18–2.62), p = 0.005], while there was no significant association for patients with CAPRA-S Score 6–12 [n = 86, HR = 1.38 (95% CI = 0.81–2.355), p = 0.235].

### Multivariate analyses

Results from a multivariate model of clinicopathological variables and miR-205 vs BF for all patients are presented in Table 2. In addition to the clinicopathological factors CAPRA-S Score (p < 0.001) and perineural infiltration (p = 0.001), a high expression of miR-205 correlates to a worse BF (HR = 1.70, p = 0.001). Clinicopathological factors associated to CF and PCD in our cohort are previously reported^19^. When further exploring which clinicopathological subgroups the miR-205 expression had prognostic value, a multivariate model stratified on ISUP Grade was calculated and is presented in Table 3. For ISUP Grade 1–2, the only significant prognostic factors associated with increased BF were perineural infiltration (HR = 1.93, p = 0.003) and high miR-205 expression (HR 2.07, p = 0.001). Regarding ISUP Grade 3–5, the only factor associated with increased BF was pT-stage (p < 0.001).

**Table 2:** Prognostic factors and their independent associations with biochemical failure in prostate tissue in 535 prostate cancer patients (multivariate analyses; Cox regression with backward conditional model).

Characteristics	Patients		Biochemical failure	
	(n)	(%)	HR (95% CI)	p
**CAPRA-S Score**				**<0.001**
0–2	169	32	1	
3–5	258	48	1.83 (1.19–2.81)	
6–12	102	19	4.55 (2.88–7.20)	
Missing	6	1	—	
**Tumor size**				**NS**
0–20 mm	250	47		
>20 mm	285	53		
**Perineural infiltration**				**0.001**
No	250	47	1	
Yes	285	53	1.78 (1.27–6.93)	
**Lymphovascular infiltration**				**NS**
No	492	92		
Yes	43	8		
**miR-205 in epithelium**				**0.001**
Low expression	220	41	1	
High expression	245	46	1.70 (1.23–2.35)	
Missing	70	13	—	

Abbreviations: HR = hazard ratio; NS = not significant and removed by backward model before last step of analyses.

**Table 3:** Prognostic factors and their independent associations with biochemical failure in prostate tissue in 535 prostate cancer patients (multivariate analyses; stratified Cox regression with backward conditional model).

Characteristics	Patients		Biochemical failure			
			ISUP Grade 1–2		ISUP Grade 3–5	
	(n)	(%)	HR (95% CI)	p	HR (95% CI)	p
**pT-stage**				NS		<**0.001**
pT2	374	70			1	
pT3a	114	21			2.78 (1.47–5.25)	
pT3b	47	9			4.99 (2.66–9.35)	
**Preop PSA**				NS		NS
PSA < 10	308	57				
PSA > 10	221	42				
Missing	6	1				
**Positive surgical margin**				NS		NS
No	249	47				
Yes	286	53				
**Tumor size**				NS		NS
0–20 mm	250	47				
>20 mm	285	53				
**Perineural infiltration**				**0.003**		NS
No	250	47	1			
Yes	285	53	1.93 (1.25–2.97)			
**Lymphovascular infiltration**				NS		NS
No	492	92				
Yes	43	8				
**miR-205 in epithelium**				**0.001**		NS
Low expression	220	41	1			
High expression	245	46	2.07 (1.37–3.15)			
Missing	70	13	—			

Abbreviations: HR = hazard ratio; NS = not significant and removed by backward model before last step of analyses.

## Discussion

We found no association between PC relapse and miR-205 expression assessed in tumor epithelium of PC patients treated by radical prostatectomy. However, we demonstrate that high expression of miR-205 in normal prostate epithelium is independently and significantly associated with biochemical recurrence. Interestingly, our findings raise the hypothesis of the potential impact of normal epithelium in prostate tumors. There was a significantly higher mean expression of miR-205 in normal epithelium compared to tumor epithelium, confirming results from previous studies. We found no association between miR-205 expression and CF or PCD, possibly due to a low number of events in these subgroups.

To our knowledge, this is, hitherto, the largest study of miR-205 expression vs clinical outcome in PC patients. The strengths of our study are the size of the multicenter cohort, the long clinical follow-up, and the *in-situ* examination in both normal and tumor epithelium and stroma. Although the ISH technique is labor-intensive, its strength compared to the widely used real-time polymerase chain reaction (RT-PCR) technique, is the ability to assess marker expressions in the different tissue compartments and cell types. In PC this is highly attractive due to the multifocal nature of the tumor tissue. Despite the long clinical follow-up (mean 12.4 years), the relatively low incidence of clinical recurrence and prostate cancer-specific death leaves a relatively low number of events. This demonstrates the need for even larger PC studies with longer follow-up to properly evaluate these endpoints. Other weaknesses are the retrospective design and that this study is inherently biased towards the selected group of patients that are considered healthy enough to undergo prostatectomy and towards stages of PC which are perceived surgically curable.

Several previous studies have consistently characterized miR-205 as a tumor suppressor generally downregulated in PC^13–15,32^. However, these studies were based on patients with higher histological grades of cancer in contrast to our patients’ localized disease. Hulf *et al*., however, showed that epigenetic-induced repression of miR-205 is associated with worse prognosis, validated by a cohort with localized PC cases consisting of 149 patients who had had a radical prostatectomy performed^33^. They found overexpression of miR-205 in PC cells to negatively affect cell viability, consistent with a tumor suppressor function.

We found that miR-205 was less expressed in tumor epithelium compared to normal epithelium, consistent with previous studies^13–15,17,32–35^. In line with this, we did not find any association between miR-205 expression in tumor epithelium and clinical outcome. Surprisingly, the prognostic impact of miR-205 was exclusively related to the normal prostate epithelium in PC patients. Initially, our data appeared contradicting as the active tissues in the carcinogenic processes traditionally has been considered to be tumor epithelium and stromal cells. Studies of the interplay between normal morphological and neoplastic epithelial cells have been limited. Hence, little is known about the function of normal epithelium in tumorigenesis. However, a few recent studies have revealed that normal epithelial cells, in addition to normal cells surrounding the tumor, can exert an anti-tumor activity on prostate carcinoma cells. Trevino *et al*. proposed that normal epithelial cells have the potential to revert some of the traits of tumor cells, effectively normalizing the phenotypic characteristics of the tumor cells^34,36^. The integrity and homeostasis of the epithelium are of vital importance to survival. These processes are maintained during growth and in response to damage by the evolvement of defensive mechanisms^37^. This suggests that normal epithelium may have a more important role in controlling tumor expansion than previously acknowledged, although crosstalk between normal and neoplastic epithelial cells is not fully understood.

Based on the studies cited above and our presented results, we hypothesize that the morphologically normal epithelial cells in PC specimens are potentially active functional cellular constituents counteracting the carcinogenic processes of tumor cells, in which one of the counteracting mechanisms might be expression of the tumor suppressor miR-205 in low and intermediate grade tumors. In our cohort, the prognostic importance of miR-205 expression was primarily found in low-risk cancers such as ISUP Grade Group 1–2 (Gleason 3 + 3 and 3 + 4) and CAPRA-S Score < 6. A possible mechanism why low-risk cancers with high expression of miR-205 in the normal epithelium are more prone to BF, might be that these tumors inhabit properties to recur, while tumors lacking this ability does not induce protective upregulation of miR-205 in normal epithelium to prevent development of tumor aggressiveness. For the more advanced tumors (ISUP Grade ≥3, CAPRA-S Score ≥6), one may postulate that the tumor cells have overcome the influence of the normal epithelium and have inhibited or bypassed their tumor suppressive properties. Thereby, high miR-205 expression in the normal epithelium may be a marker of the normal epithelium’s efforts to prevent more aggressive tumors to develop. It has been suggested that normal epithelial cells, secreting tumor suppressive factors such as Il-6^38^, TNFα^39,40^ and TGFβ1^39^, play an important role in influencing the molecular and physiological state of tumor cells^36,41^. In support of our hypothesis, recent studies suggest that, at the initial phase of tumor expansion, normal epithelium may provide a tumor suppressive environment which cancer cells need to overcome to cause tumor progression^34,36,41^.

Our findings are also supported by Kalogirou *et al*. They found a consistent tendency for miR-205 upregulation to correlate with an adverse outcome of PC patients, supporting our findings of an association between high expression of miR-205 and BF^14^. They suggested that miR-205 expression might be tightly controlled at different tumor stages, affecting the expression of either tumor suppressors or oncogenes. However, their RT-PCR study could not differentiate between expressions in normal or tumor epithelium, nor epithelium or stroma. Gandellini *et al*. found pathological loss of miR-205 in PC to favor tumorigenesis by creating discontinuities in the basal membrane, and demonstrated that therapeutic replacement of miR-205 can restore basal membrane deposition, thus hampering cancer progression^17^. In a later study, they found miR-205 to prevent the malignant interplay between prostate cancer cells and associated fibroblasts^35^, supporting our hypothesis.

We also observed a positive correlation between miR-205 and VEGF-A/VEGFR-2. It has previously been shown that high expression of VEGF-A and VEGFR-2 is correlated to BF and CF in prostatectomy patients^30^. miR-205 directly targets VEGF-A, functioning as a tumor suppressor in breast cancer^42^. No studies have, to our knowledge, assessed associations between PDGFs and miR-205. However, we found a strong correlation between PDGF-D and miR-205, whereas PDGF-B and PDGFR-β correlated to miR-205 to a less extent as well.

In conclusion, our results add support to the potential prognostic role of normal epithelium in PC and its potential crosstalk to surrounding tissues.

As high miR-205 expression in normal epithelium was an overall predictor for BF for patients with localized disease, we propose normal epithelium acts to hinder further aggressiveness in the more aggressive low-grade tumors. This can be by exerting tumor suppressor effects of miR-205 in low- and intermediate grade PC tumors. Although speculative, this intriguing postulation mandates further research to clarify the mechanisms of crosstalk between tissues. Considerable resources are currently being invested in the development of miR anti-cancer therapy. However, the success of such specific therapeutic targeting will rely on a deeper understanding of the biological mechanics at play.

### Equipment and settings

Figure 1 is edited in Adobe Photoshop for cropping, while frames and shadows are made in Microsoft Excel. No adjustments to color, brightness or contrast were done. The subpanels of Fig. 2 are exported from SPSS and joined in Microsoft Paint. All figures and tables are made by the first author, Y.N.

### Availability of materials and data

The datasets generated during and analysed during the current study are not publicly available in respect to patients’ privacy.
